# Cold-Stress Response of Probiotic *Lactobacillus plantarum* K25 by iTRAQ Proteomic Analysis

**DOI:** 10.4014/jmb.1909.09021

**Published:** 2019-11-22

**Authors:** Shaoli Liu, Yimiao Ma, Yi Zheng, Wen Zhao, Xiao Zhao, Tianqi Luo, Jian Zhang, Zhennai Yang

**Affiliations:** 1 Beijing Advanced Innovation Center for Food Nutrition and Human Health, Beijing Technology and Business University Beijing, P.R. China; 2 Engineering and Technology Research Center of Food Additives, Beijing Technology and Business University, Beijing, 100048, P.R. China

**Keywords:** Proteomics, iTRAQ, *Lactobacillus plantarum* K25, cold tolerance

## Abstract

To understand the molecular mechanism involved in the survivability of cold-tolerant lactic acid bacteria was of great significance in food processing, since these bacteria play a key role in a variety of low-temperature fermented foods. In this study, the cold-stress response of probiotic *Lactobacillus plantarum* K25 isolated from Tibetan kefir grains was analyzed by iTRAQ proteomic method. By comparing differentially expressed (DE) protein profiles of the strain incubated at 10°C and 37°C, 506 DE proteins were identified. The DE proteins involved in carbohydrate, amino acid and fatty acid biosynthesis and metabolism were significantly down-regulated, leading to a specific energy conservation survival mode. The DE proteins related to DNA repair, transcription and translation were up-regulated, implicating change of gene expression and more protein biosynthesis needed in response to cold stress. In addition, two-component system, quorum sensing and ABC (ATP-binding cassette) transporters also participated in cell cold-adaptation process. These findings provide novel insight into the cold-resistance mechanism in *L. plantarum* with potential application in low temperature fermented or preserved foods.

## Introduction

Lactic acid bacteria (LAB) play important roles in food fermentation in providing positive attributes to food sensory properties, quality and safety [1]. During fermentation and storage periods, these bacteria may encounter different stress conditions, *e.g.* low temperature, low pH, high ethanol concentration, high osmotic pressure and high salt stress, etc. [2, 3]. Traditional food fermentation is often executed under relative coldness conditions (0-10°C), *e.g.* Korean kimchi, pickled cabbage, fermented bean curd and cucumber, etc., where there is presence of cold-resistant LAB strains surviving the cold stress conditions. In fermented ice cream, rigorous low temperature aging, freezing and preserving are experienced by LAB and the tolerant strains may survive during the production processes [2]. Although numerous LAB have been identified and utilized from traditional fermented products, the cold-resistant LAB have long been used without being fully understood.

It was known that cold-resistant LAB could adapt to a cold environment, maintaining cell viability and producing more lactic acid to enhance product flavor and health properties [3]. However, cold stress would cause changes in membrane fluidity (influencing nutrient transport), lowering of enzyme activity (influencing cellular metabolism) and RNA structure stability (influencing translation process), all of which further influence the bacteria viability [4]. During the past few decades, there have been numerous studies exploring cold resistance mechanisms in pathogenic bacteria such as *Listeria monocytogenes*, *Cronobacter turicensis*, *Staphylococcus aureus*, *Escherichia coli* and environmental microorganisms such as *Cryptococcus humicola* and *Desulfovibrio vulgaris*, as they could survive during low temperature preservation and pose a great threat to food safety, whereas only a few studies have focused on LAB [5-9]. The main opinions about cold resistance mechanisms have mainly been related to changes in cell morphology, decreased membrane fluidity and increased integrity, modified carbohydrate and amino acid biosynthesis metabolism, and increased RNA degradation rate to counterbalance the RNA stabilization [10]. Numerous chaperones and transcription regulators also play important roles since the cold shock protein family has been identified as a main factor in cold adaptation [11]. Due to their wide potential application, further investigation into the cold-adaptation mechanisms of LAB may lead to discovery of new agents or methods that might also facilitate the adaptation and growth of LAB at low temperature.

*Lactobacillus plantarum* is commonly found in various environment niches, including those under extreme conditions such as low pH, high osmotic pressure and low temperature, etc., where there is presence of other tolerant strains adapting to the stress conditions. Previously, *L. plantarum* K25 isolated from low temperature preserved Tibetan Kefir grains was characterized as a probiotic strain [12]. In this study, the growth characteristics of *L. plantarum* K25 at low temperature were investigated, and the molecular mechanism involved in the cold adaptation of the strain was studied by iTRAQ proteomic analysis by comparing the protein profiles expressed by the strain at 10°C and 37°C, respectively. The temperature at 10°C was used to mimic the cold fermentation and storage condition. The protein samples were prepared from the strain at its optimal growth in this study. The present study may provide further insight into the cold-adaptation strategy employed by *L. plantarum* under cold fermentation and storage conditions.

## Materials and Methods

### Bacterial Strain and Growth Conditions

*L. plantarum* K25 was provided by Jilin Academy of Agricultural Science of China. It was isolated from Tibetan Kefir with antioxidant and gut microbiota regulating properties [13, 14]. *Lactobacillus rhamnosus* GG (LGG, ATCC: 53103) obtained from the LAB collection in the authors’ lab were used for comparison of growth at low temperature. For studies on cold response, these strains were grown in MRS medium at 10°C and 37°C, respectively. At 0, 2, 4, 8, 10, 12, 16, 24, 36, 48, 60, 72, 84, 96 h after inoculating, the medium was determined for absorbance of OD_600nm_ by spectro-photometer. At the corresponding time point, pH was detected respectively. Each growth experiment was repeated three times.

### Protein Extraction and Quantification

Bacterial cell samples were prepared by centrifugation of the culture samples obtained from the optimal growth (about the end of the logarithmic growth phase) of L.plantarum K25 at 10°C and 37°C, respectively, and a sample of the latter was used as control. Three independent replicates were collected for each sample. The cell pellets obtained from centrifugation of the bacterial culture were resuspended and washed 3 times with PBS (phosphate-buffered saline). The lysis solution (1 ml of lysis buffer contained 1 µl protease inhibitor, 10 µl of 1 M DTT and 10 µl of 100 mM PMSF) was added to one-fifth of the original volume of the bacterial cultures, and the mixture was incubated at 4°C for 10 min. The cells were further lysed by ultra-sonication and centrifuged at 14,000 ×*g* for 15 min at 4°C. Supernatant was collected and the protein concentration was quantified using the Bradford method.

### Protein Digestion and iTRAQ Labeling

Approximately 200 µg of protein was mixed with 4 µL DTT and incubated at 37°C for 1 h. Then the mixture was added with 20 µl iodoacetamide and incubated for 0.5-1 h at room temperature. The alkylated protein was collected by centrifugation at 12,000 ×*g* for 20 min. Subsequently, the protein was dissolved with 120 µl solution (8 M urea and 100 ml Tris-HCl) and centrifuged at 12,000 ×*g* for 10 min, and this procedure was repeated twice. The supernatant was added with 100 µl TEAB buffer solution and centrifuged at 12,000 ×*g* for 10 min. After washing three times, the pellet was digested with 4 µl trypsin solution at 37°C overnight. The digested samples were then centrifuged at 12,000 ×*g* for 10 min, and the supernatant was discarded. iTRAQ labeling was done by using an iTRAQ Reagent 8Plex Multiplex Kit (4381663, Sigma-Aldrich, USA) according to the manufacturer’s instructions. iTRAQ reagent was used to coordinately label the peptides from the control samples (37°C) and the experimental samples (10°C). The labeled peptides were incubated at room temperature for 1 h and reaction was stopped by adding 50-100 µl of ultrapure water. Then, 1 µl of sample was removed from each group and mixed to confirm that the samples had been labeled successfully. Finally, the labeled peptides were pooled and lyophilized.

### LC-MS/MS Analysis and Database Search

The peptide sample (100 µg) was dissolved with 500 µl NH_4_HCO_3_, and eluted with a Strata-X C18 column using 1 ml 0.1% FA and 8%acetonitrile, and 1 ml eluate liquid was collected and lyophilized. The dried sample was re-dissolved in 20 µl 0.5 M TEAB and divided into 12 fractions. The analytical separation was performed using an Eksigent Chromxp Trap Column (350 µm × 0.5 nm, 120A, 3 µm) and a pulled quartz tip as the emitter (New Objectives, USA). LC was performed with a flow rate of 0.3 ml/min using the following elution program: 0-65min: 95% buffer A (0.1% formic acid+ 5% acetonitrile) + 5% buffer B (0.1% formic acid + 95%acetonitrile); 65-70 min: 70% buffer A +30% buffer B; 70-80 min: 50% buffer A+ 50% buffer B; 80-85 min: 20% buffer A+ 80% buffer B; 85-90 min: 95% buffer A+ 5% buffer B.

### Data Analysis

The original data files gathered by MS in wild format were processed with Protein Pilot Software V4.5 (AB Sciex, USA) against the *L. plantarum* K25 protein database using the Paragon algorithm. For protein identification, a mass tolerance of 0.05 Da was permitted for intact peptide masses and 0.1 Da for fragmented masses, with allowance for one missed cleavage upon trypsin digest. Several parameters in Mascot were set for peptide searching, including iTRAQ quantification (N-term, K), cysteine modified with iodoacetamide, and trypsin digestion. A protein containing at least two unique peptides was required for quantitation. The quantitation protein ratios were weighted and normalized by the median ratio in Mascot. Protein with at least 1.5-fold change between two samples and a *p* value <0.05 was considered as one significantly differential species. COG (Cluster of Orthologous Groups of proteins) analysis was conducted according to the NCBI database (http://www.ncbi.nlm.nih.gov/COG/).

### RNA Extraction and Quantitative Transcriptional Analysis

All gene manipulation was based on the genome of *L. plantarum* K25 in NCBI. RNA was extracted using a Universal RNA Extraction Kit (TianGen, China) according to the instructions. RNA yield and quality were evaluated with a UV spectrometer Q5000 (Quwell). Then, approximately 4 ul of RNA was reverse transcribed with a Tastline Cell DNA Kit (TianGen). Quantitative real-time PCR (qRT-PCR) was performed using the Real Master Mix (TianGen) with a final volume of 25 ul in each reaction system, which included 12.5 ul Mix containing SYBR Green 1, dNTPs, AmpliTaq Gold Fast DNA polymerase LD, 10x buffer, and 1 ul cDNA, 0.5 ul of each primer and 10.5 ul of ddH_2_0, the target genes involved in the genome sequence of *L. plantarum* K25 and primers (designed by Primer Primer 5.0). The amplification procedure included: denaturation at 94°C for 2 min; 35 cycles of 94°C for 30 sec; 53°C for 30 sec; 72°C for 30 sec, and 72°C for 10 min. At the end of PCR cycles, melting curve analyses were performed using the LightCycle Nano qRT-PCR system. All the samples were produced and run in triplicate, and 16S rRNA gene was used as the reference. The primer sequences for the representative proteins highly differentially expressed were listed in Table S1.

## Results

### Growth Behavior under Low Temperature Condition

**Fig. 1:**
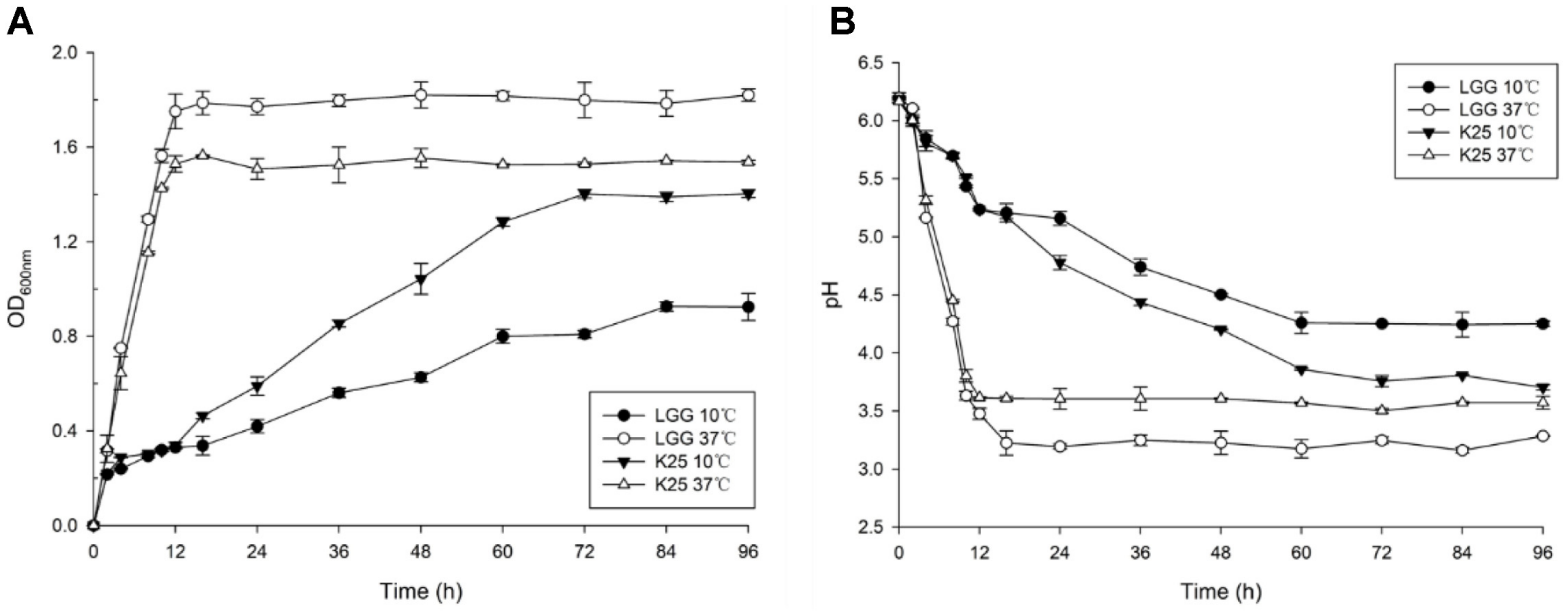
Growth behavior of *L. plantarum* K25 and *L. rhamnusus* GG at 10°C and 37°C in the MRS medium as monitored by measurement of OD_600nm_ (**A**) and pH (**B**).

Rather slow growth of *L. plantarum* K25 was observed at 10°C in comparison with its relatively fast growth at 37°C as shown by the significantly prolonged and delayed logarithmic growth phase at the low temperature (Fig. 1A). It took about 72 h for strain K25 to reach stationary growth at 10°C, and took about 14 h at 37°C. Similarly, LGG also grew more slowly at 10°C than at 37°C. However, strain K25 (OD_600nm_ 1.4159 at 72 h) showed better growth than strain LGG (OD_600nm_ 0.8497 at 72 h) at the low temperature. The change of pH value correlated well to that of OD_600_ at the corresponding temperature of growth with the pH values of 3.97 and 4.25 at 72 h at 10°C for strains K25 and LGG, respectively (Fig. 1B). The results indicated that both strains of K25 and LGG might develop a mechanism to adapt to the long-term low temperature condition, though they exhibited different degrees of tolerance to the low temperature. In the following study, *L. plantarum* K25 was selected for proteomic analysis of its response to low temperature (10°C) stress in comparison with that to its normal growth condition (37°C).

### Primary Data Analysis and Protein Identification

**Fig. 2:**
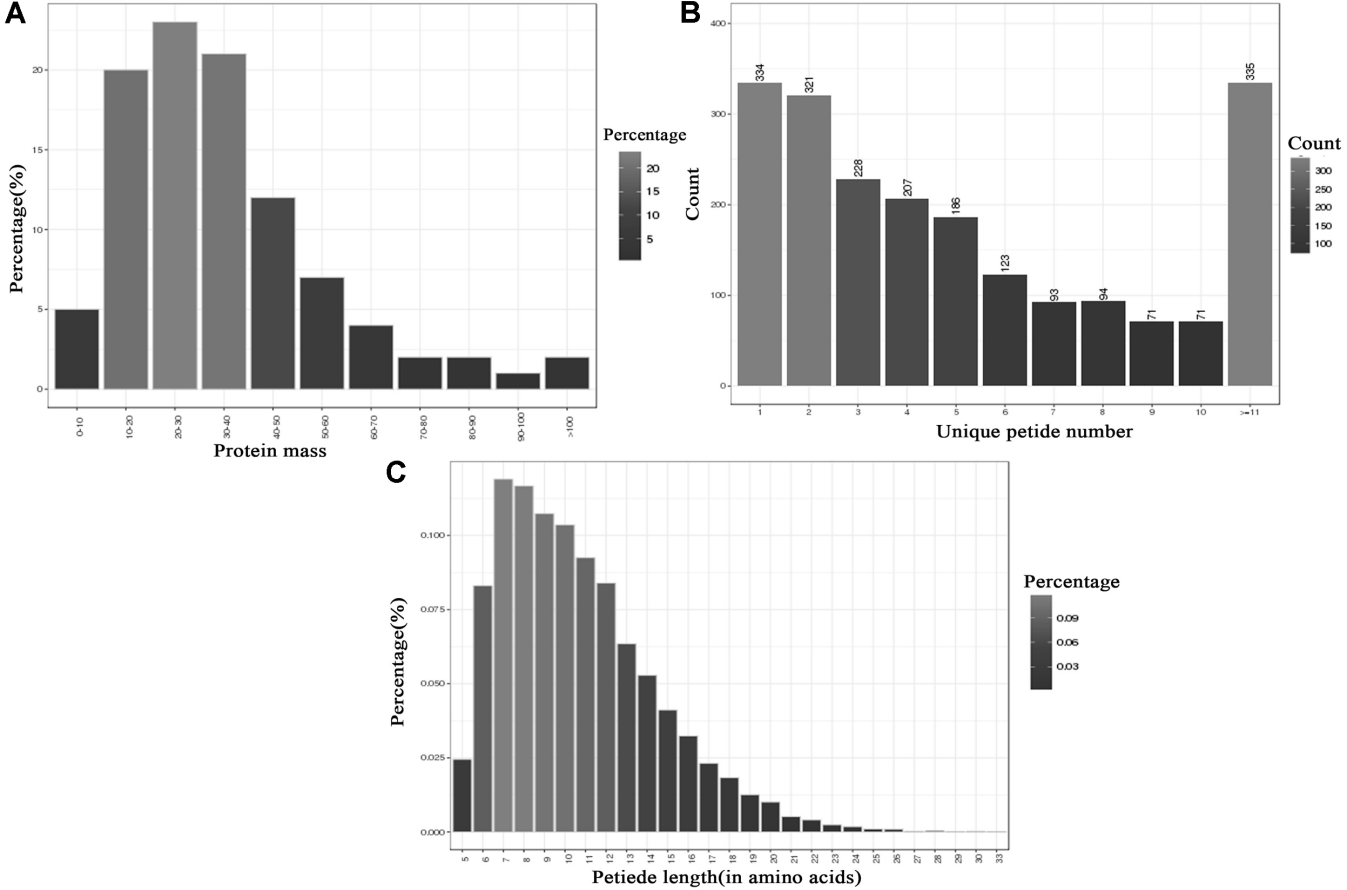
Identification and analysis of the *L. plantarum* K25 proteome. (**A**) Identified proteins were based on their protein mass. (**B**) Numbers of unique peptides that match to proteins. (**C**) Percentages of peptide length (in amino acids).

It is significant to explore the cold-adaptation mechanism for LAB involved in food processing at low temperature. Using iTRAQ-based technology in our study enabled us to further extend the search scope for differentially expressed proteins at low temperature when compared with traditional 2-DE proteome studies. Among total of 227,107 spectra obtained, 49,106 spectra were identified including 12,483 unique peptides and 2,063 identified proteins before grouping with a 1% false discovery rate (FDR) as the cutoff in two independent experiments. There were 83%, 15% and 2% of proteins with molecular mass of 0-50 kDa, 50-100 kDa and >100 kDa, respectively (Fig.2A). The distribution of peptide numbers is shown in Fig. 2B. These proteins with single peptide, 2-4 peptides, 5-7 peptides, 8-10 peptides and above 11 peptides consisted of 334, 756, 392, 236 and 355, respectively. A total 83.89% of proteins contained at least 2 peptides. Unique peptide length was between 5 and 72 (in amino acids) (Fig. 2C).

### Analysis and Functional Annotation of Differential Proteins

**Fig. 3:**
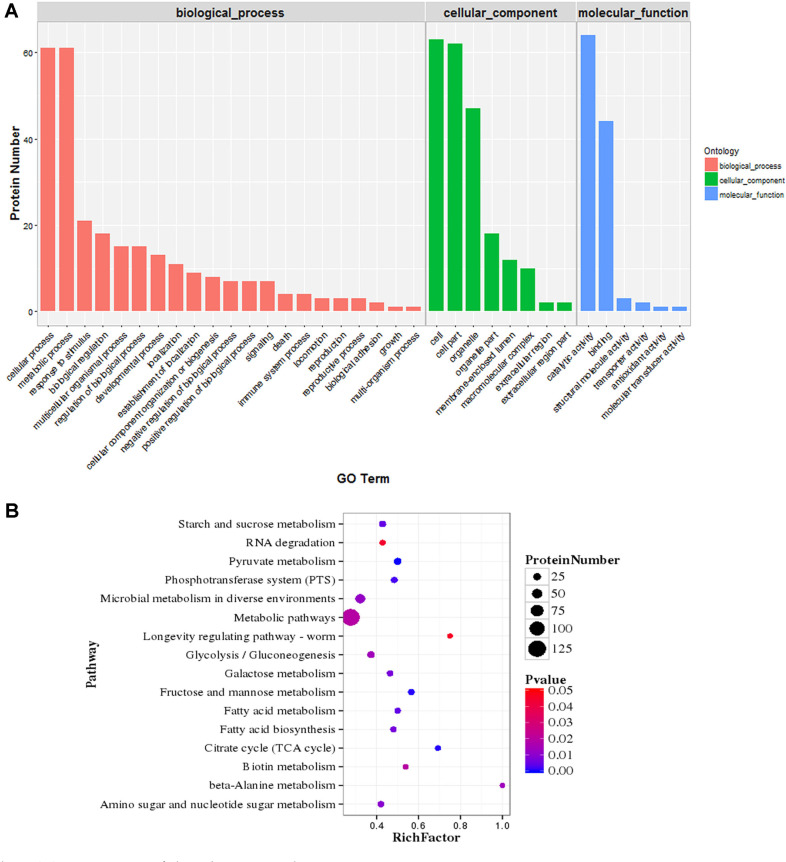
GO and KEGG annotation of distribution analysis. (**A**) Functional categorization of identified proteins according to the Gene Ontology Consortium classification. The vertical scales are protein numbers. Blue bars refer to DE proteins involved in molecular component; Green bars refer to DE proteins in cellular component; Pink bars refer to DE proteins involved in biological process. (**B**) Pathway classification based on KEGG enrichment analysis of differentially expressed proteins of *L. plantarum* K25 in response to cold stress. Rich factor, the ratio of the number of differentially expressed genes to the number of total genes in this pathway. Proteins with at least 1.5-fold change are shown; adjusted *p* ≤ 0.05 for all data selected.

A total of 509 common DE proteins were found. The DE proteins and details about their functions were summarized in Table S2. A Gene Ontology (GO) enrichment analysis was performed to classify the differentially expressed proteins according to their biological process, location in cellular component, and molecular function. Functional assignment for each protein was made using the nomenclature developed for clusters of orthologous groups of proteins (COG). In the category of cellular components, the DE proteins were mainly located in cell organelle and membrane; in terms of molecular function, the DE proteins mainly executed catalytic and binding activity; for biological processes, the DE proteins were mainly involved in cellular process, metabolic process, response to stimulus and biological regulation (Fig. 3A).

The KEGG pathway enrichment analysis was conducted to determine the biological pathway to which the DE proteins belonged (Fig. 3B). In the different temperature environments, 29 DE proteins were involved in carbon metabolism, such as pyruvate metabolism (path: ko00620), glycolysis/gluconeogenesis (path: ko00010) and citrate acid cycle (TCA) (path: ko00020), indicating that carbohydrate metabolism might be the main factor influencing cell survival at 10°C. In comparison, only 16 DE proteins were related to amino acid metabolism and biosynthesis, including beta-alanine metabolism (path: ko00410), lysine biosynthesis (path: ko00300), and histidine metabolism (path: ko00340). There were 14 DE proteins involved in fatty acid metabolism (path: ko01212) and biosynthesis (path: ko00061). Ribosome and protein metabolism might be the critical biological process for cell viability. There were approximately 55 DE proteins involved in transcription and translation processes, such as RNA degradation (path: ko03018), protein export (path: ko03060), ribosome (path: ko03010). Additionally, proteins involved in peptidoglycan biosynthesis (path: ko00550), two-component system (path: ko02020), ABC transporters (path: ko02010) and phosphotransferase system (PTS) (path: ko02060) were also differently expressed. Further functions of these differently expressed proteins are discussed in the following section.

### Transcriptional Expression Analysis of Selected Proteins as Revealed by qRT-PCR 

**Fig. 5:**
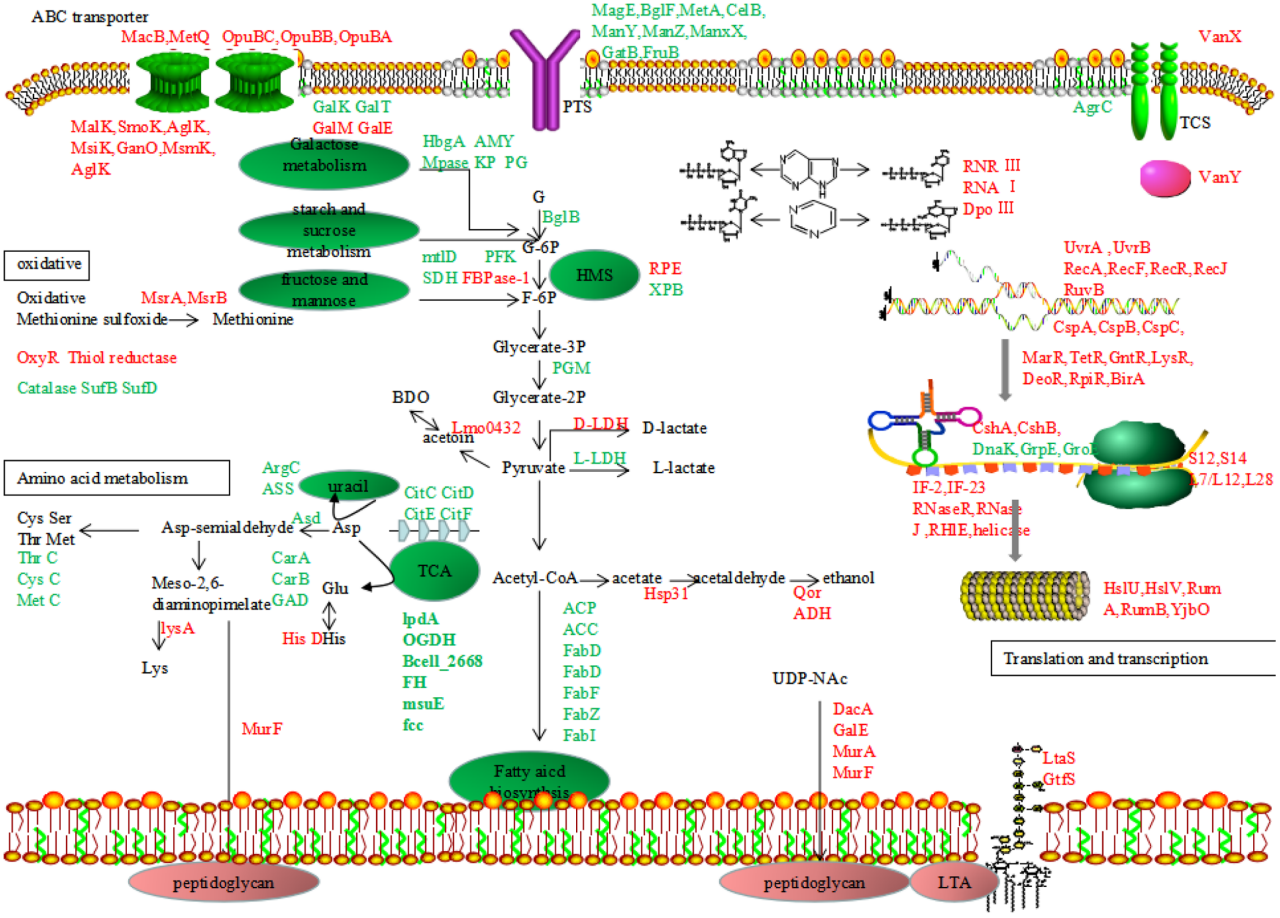
Proposed model for response mechanisms of *L. plantarum* K25 under cold stress condition. Up-regulated and down-regulated proteins (Fold change > 1.5 or <0.667, *p* < 0.05) are presented in the color of red and green, respectively.

To confirm the accuracy of the iTRAQ ratios, correlation between some representative proteins with their cognate gene expressions was analyzed. The results were shown as the mean fold changes standard deviation, and significant differences were determined using one-way ANOVA (*p* < 0.05). As shown in Fig. 5, the mRNA levels of cspA, cshA, fanD, groEL, fruB were correlated well with the iTRAQ proteomic results. However, expression of genes hsp31 and mreB was significantly up-regulated, and gene dnaK significantly down-regulated, probably due to complex translational regulations in *L. plantarum*. The overall trend between the gene and protein expression level was consistent, indicating the credibility of the iTRAQ ratios in the present study.

## Discussions

Lowering temperature may decrease bacterial growth and proliferation. Meanwhile, the bacteria stimulate self-adjusting to develop cold-resistance mechanisms. The results of the present study revealed that *L. plantarum* K25 displayed a complex biological network to tackle cold-stress mainly by adjusting carbohydrate, amino acid and fatty acid metabolism and biosynthesis. The cold-resistant mechanism of this strain involved a specific energy conservation, survival mode and repairing system, and enhanced protein synthesis ability. Relevant chaperons and transcription regulators against cold stress were also identified.

In this study, we found that 29 DE proteins were involved in carbohydrate metabolism and energy production and conversion including 6 proteins up-regulated and 23 proteins down-regulated, indicating that metabolic nutrient intake and energy production were inhibited to reduce the metabolic level and growth rate at low temperature. These DE proteins participated in glycolysis/glucoenogensis, citrate cycle (TCA), pentose phosphate pathway (PPP), starch and sucrose metabolism, fructose and mannose metabolism and pyruvate metabolism. Down-regulated proteins indicated that during cold-stress condition, bacteria strictly inhibited intake of fructose and mannose which might be needed for bacterial growth. In addition, there was decreased expression of DE proteins involved in sucrose, galactose and cellulose metabolism, which indicated repression of carbohydrate metabolism under low temperature condition. Similar decrease in expression of proteins related to carbohydrate metabolism in L. kefiranofaciens M1 and *L. sakei* in response to cold-stress condition was reported earlier [15, 16].

A large group of proteins with different concentrations was related to amino acid biosynthesis and metabolism. Histidinol dehydrogenase (lp-2172) (responsible for histidine biosynthesis) was upregulated 1.827-fold. Histidine is an amino acid that has various biological functions such as supplying materials for protein synthesis, being a precursor of physiologically activate substrates and regulating hormone secretion and cell turnover [17]. Besides, we observed down-regulation of the chill-repress protein was involved in the arginine biosynthesis and catalysis of the urea cycle initial step, respectively, using arginine as a substrate for ammonia generation. Down-regulation of carbamoyl phosphate synthase large subunit (lp-2284), carbamoyl phosphate synthase small subunit (lp-2285) (catalysis of the transformation of L-glutamine and carbamoyl phosphate) resulted in accumulation of glutamate and glutamine in cytoplasm which can be used as osmotic protectants to enhance cell survivability and relieve oxidative damage [18]. Up-regulation of diaminopimelate decarboxylase (lp-1432) to increase its cellular abundance promoted transformation of meso-2,6-diaminopimelate into lysine. The intermediates formed in the lysine biosynthesis pathway were also required for peptidoglycan biosynthesis. Secretion of both peptidase C69 (lp-0312, lp-0223) and Xaa-Pro aminopeptidase (lp-1326) increased at 10°C, suggesting that *L. plantarum* K25 would scavenge extracellular nitrogen and carbon sources after exposure to an oligotrophic environment [19].

Fatty acid biosynthesis was negatively affected in *L. plantarum* K25 under cold-stress condition. These results indicated that cold stress would cause *L. plantarum* K25 to reduce membrane fluidity and lead to an inefficient membrane-associated function (*e.g.* active transport and protein secretion). Besides, down-regulation of proteins involved in fatty acid metabolism also decreased energy production, suggesting a mechanism of energy saving in *L. plantarum* K25 for its survival under cold-stress condition. As the cell wall is the first line of defense against environment stress for bacteria, the enhancement of cell wall component biosynthesis may be a self-protective mechanism of *L. plantarum* K25 in response to cold. Some proteins were up-regulated to increase total peptidoglycan production. It was noted that peptidoglycan biosynthesis in *S. aureus* cell wall also increased after prolonged cold exposure [20].

In this study, numerous chaperons were identified in *L. plantarum* K25 at low temperature. These proteins could be treated as cellular garbage and undergo proteolysis with aid of specific chaperons [21].The Csp (cold shock protein) family such as CspA, CspB and CspC could respond to cold shock and act as RNA chaperons to improve the protein synthesis capacity by specifically binding to single-stranded nucleic acids, thereby reducing formation of mRNA secondary structure [22]. Therefore, these proteins were essential for effective protein synthesis and cell viability at low temperature.

Structural integrity of DNA molecules is sensitive to changes in the physiological environment. Therefore, the enhancement of protein expression associated with DNA repair, recombination and stabilization in *L. plantarum* K25 exposed to cold stress indicated that there might be a DNA injury after cell experienced cold stress and an excellent DNA repair was needed [23]. Beyond that, 22 DE proteins were identified as transcription factors (TFs), which were involved in numerous cellular functions, including oxidative stress responses, cell wall shape, quorum sensing, efflux pumps, secretion, motility, nitrogen fixation, virulence, cell division, metabolism and environmental recognition. These proteins regulate a wide range of cellular activities including stress response, efflux pump production, and modulation of metabolism [24, 25].

Some proteins increased their abundance upon cold shock, suggesting that these proteins might be involved in inter- or intra-cell signaling system including two-component system (TCS), ATP-binding cassette (ABC) transporter and quorum sensing (QS) [26]. Change of gene expression related to TCS and QS relied on external signaling and cell density, respectively [27, 28] Relationship between cold stress and oxidative stress was evidenced in *L. monocytogenes* at the transcription level [29], but it was rarely studied in lactic acid bacteria. It was known that under cold stress condition, solubility of gasses and production of toxic reactive oxygen species (ROS) increased significantly, thus triggering expression of specific proteins to protect cell machinery from oxidative damage.

In conclusion, the molecular mechanism of the cold-stress response of probiotic *L. plantarum* K25 was investigated by comparative and functional proteomic analysis. A total of 2,063 proteins were identified, and 506 proteins were found to be differentially expressed. By analyzing the critical DE proteins, *L. plantarum* K25 was revealed to employ a cold-adaptation mechanism mainly by regulating carbohydrate metabolism and energy production, fatty acid biosynthesis, and amino acid biosynthesis, and the proposed molecular mechanism of *L. plantarum* K25 in response to cold stress based on the results of this study was diagramed in Fig.5. Under cold-stress condition at 10°C, carbohydrate metabolism was inhibited to reduce bacterial growth rate and save energy for survival. Decreased fatty acid biosynthesis and metabolism led to change of membrane composition and reduced cell membrane fluidity, while increased biosynthesis of peptidoglycan and LTA significantly enhanced cell wall integrity to protect cells from environmental injury. Furthermore, improved purine and pyrimidine metabolism provided more substrates for DNA biosynthesis, thus increasing transcription and translation efficiency. There were also many chaperons and transcription factors differentially expressed to assist in precise and efficient synthesis of proteins needed for counteracting cold stress. The present study provided further insight into the basic physiology of *L. plantarum* responding to cold stress, facilitating development of novel strategies to increase bacterial survivability at low temperature.

## Supplemental Materials



Supplementary data for this paper are available on-line only at http://jmb.or.kr.

## Figures and Tables

**Fig. 4:**
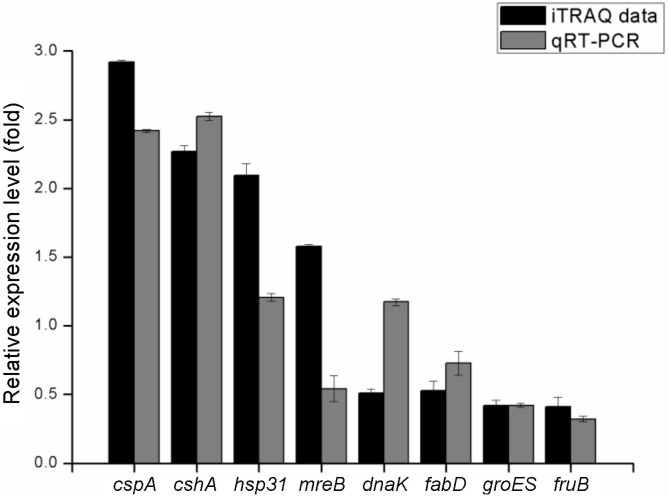
Comparative analysis of the transcripts (mRNA levels) and protein levels of the representative differentially expressed proteins revealed by qRT-PCR and iTRAQ, respectively, including up-regulated proteins (*cspA, cshA, hsp31, mreB*) and down-regulated proteins (*dnaK, fabD, groES, fruB*).
