# Supplementary material for: Cold-Stress Response of Probiotic Lactobacillus plantarum K25 by iTRAQ Proteomic Analysis
Source: J Microbiol Biotechnol. 2019 Nov 22;30(2):187–95. doi: 10.4014/jmb.1909.09021 (PMC9728241; doi:10.4014/jmb.1909.09021)
Supplement: Supplementary file 1 [file JMB-30-2-187-supple.pdf]

J. Microbiol. Biotechnol.
https://doi.org/10.4014/jmb.1909.09021

jmb

	Protein-ID	Gene name	Primer	Fold-change
1	Lb.plantarum_K25GM000048	Csp	CCGGTTCGGATAATAAAGATG<br>CAACATTCGTTGCTTGAGGG	2.9205
2	Lb.plantarum_K25GM000450	cshA	CAAGTCGTTTACGGTGGTGC<br>GCATAGTTTGGCGTTCATCC	2.2695
3	Lb.plantarum_K25GM000090	hsp31	TAGTCCTAATGGTGGCTTCG<br>GCTTGTAACTCAGGGTTGTCC	2.093
4	Lb.plantarum_K25GM002020	mreB	AGGTGGAATCGTATCAAGTCG<br>AGTTGTTCGGCTGTGCGTT	1.5795
5	Lb.plantarum_K25GM001702	dnaK	TATTCATTCGCTCTACAACAAGC<br>ACGCTCACGGTCATCCACTA	0.51
6	Lb.plantarum_K25GM001395	fabD	CCCCAAAGCGGTAAGGTTG<br>GCAATACCAAATACTTAGCACC	0.529
7	Lb.plantarum_K25GM000638	groES	TCTGCGGGTAACTGCTGATT<br>CATACTCGCCAAGGCTCAAT	0.4205
8	Lb.plantarum_K25GM002797	fruB	AAGTGGACTGGCAAAGAATCTA<br>TACCAAGTGAAAGTGGCGTAAC	0.4105

Table S1. Primer sequences used for qRT-PCR.

Carbohydrate transport and metabolism		
Protein-ID	NCBInr Description	Ratio
Lb.plantarum_K25GM000728	Galactose mutarotase	2.091
Lb.plantarum_K25GM001558	Luciferase	1.959
Lb.plantarum_K25GM000114	Alcohol dehydrogenase	1.873
Lb.plantarum_K25GM000799	Fructose-2,6-bisphosphatase	1.762
Lb.plantarum_K25GM001028	Glycerol uptake facilitator protein	1.567
Lb.plantarum_K25GM001351	Ribulose-phosphate 3-epimerase	1.542
Lb.plantarum_K25GM001494	Phosphoglycerate mutase	0.644
Lb.plantarum_K25GM000738	Glycerol kinase	0.629
Lb.plantarum_K25GM000469	Lactate dehydrogenase, partial	0.61
Lb.plantarum_K25GM000964	Citrate lyase subunit alpha	0.608
Lb.plantarum_K25GM002987	Galactokinase	0.579
Lb.plantarum_K25GM000974	Class II fumarate hydratase	0.575
Lb.plantarum_K25GM000391	PTS cellobiose transporter subunit IIC	0.538
Lb.plantarum_K25GM001872	2-oxo acid dehydrogenase acyltransferase	0.513
Lb.plantarum_K25GM000506	PTS mannose transporter subunit IID	0.511
Lb.plantarum_K25GM002040	PTS system sorbose subfamily IIB component	0.51
Lb.plantarum_K25GM001873	2-oxoisovalerate dehydrogenase subunit beta	0.508
Lb.plantarum_K25GM001874	Pyruvate dehydrogenase (acetyl-transferring) E1 component subunit	0.5

	alpha	
Lb.plantarum_K25GM000182	Maltose phosphorylase	0.462
Lb.plantarum_K25GM001444	Maltose phosphorylase	0.459
Lb.plantarum_K25GM002352	PTS cellobiose transporter subunit IIA	0.45
Lb.plantarum_K25GM003043	Xylulose-5-phosphate phosphoketolase	0.45
Lb.plantarum_K25GM000227	Mannitol-1-phosphate 5-dehydrogenase	0.447
Lb.plantarum_K25GM000181	Sugar ABC transporter ATP-binding protein	0.446
Lb.plantarum_K25GM000185	Fructokinase	0.446
Lb.plantarum_K25GM000957	L-lactate dehydrogenase	0.421
Lb.plantarum_K25GM000960	Malate dehydrogenase	0.407
Lb.plantarum_K25GM002797	PTS fructose/mannitol specific EIIA component	0.338
Lb.plantarum_K25GM000264	PTS fructose transporter subunit IIC	0.315

Energy production and conversion		
Protein-ID	NCBInr Description	Ratio
Lb.plantarum_K25GM000772	Lactate dehydrogenase	2.935
Lb.plantarum_K25GM001545	Aldose 1-epimerase	2.554
Lb.plantarum_K25GM000091	NADPH:quinone reductase	2.177
Lb.plantarum_K25GM000975	Flavocytochrome c	0.615
Lb.plantarum_K25GM001871	Dihydrolipoyl dehydrogenase	0.585
Lb.plantarum_K25GM000710	Phosphate acetyltransferase	0.577
Lb.plantarum_K25GM002535	PTS N-acetylglucosamine transporter subunit IIABC	0.576
Lb.plantarum_K25GM000180	Alpha-amylase	0.567
Lb.plantarum_K25GM002994	Glyceraldehyde 3-phosphate reductase	0.564
Lb.plantarum_K25GM000961	[citrate (pro-3S)-lyase] ligase	0.551
Lb.plantarum_K25GM000963	Citrate (pro-3S)-lyase subunit beta	0.541
Lb.plantarum_K25GM002796	Fructose-like phosphotransferase enzyme IIB component 2	0.53
Lb.plantarum_K25GM000067	Flavoprotein	0.527
Lb.plantarum_K25GM000224	PTS mannitol transporter subunit IICB	0.526
Lb.plantarum_K25GM000962	Citrate lyase ACP	0.513
Lb.plantarum_K25GM002350	6-phospho-beta-glucosidase	0.484
Lb.plantarum_K25GM003038	Sorbitol dehydrogenase	0.481
Lb.plantarum_K25GM002353	PTS sugar transporter subunit IIB	0.477
Lb.plantarum_K25GM000504	PTS mannose transporter subunit EIIAB	0.468
Lb.plantarum_K25GM000979	NAD-dependent malic enzyme	0.464
Lb.plantarum_K25GM002865	Formate acetyltransferase	0.435
Lb.plantarum_K25GM003040	PTS galactitol transporter subunit IIB	0.419
Lb.plantarum_K25GM000975	Lactate dehydrogenase	2.935

Amino acid metabolism		
Protein-ID	NCBInr Description	Ratio
Lb.plantarum_K25GM001326	Xaa-Pro aminopeptidase	3.975
Lb.plantarum_K25GM000223	Peptidase C69	1.936

Lb.plantarum_K25GM002172	Histidinol dehydrogenase	1.827
Lb.plantarum_K25GM000312	Peptidase C69	1.758
Lb.plantarum_K25GM000346	ABC transporter ATP-binding protein	1.675
Lb.plantarum_K25GM001432	Diaminopimelate decarboxylase	1.503
Lb.plantarum_K25GM001244	Cysteine desulfurase	0.613
Lb.plantarum_K25GM002333	Threonine synthase	0.603
Lb.plantarum_K25GM002230	Cystathionine gamma-synthase	0.585
Lb.plantarum_K25GM002349	D-serine dehydratase	0.563
Lb.plantarum_K25GM002936	Glutamate decarboxylase	0.539
Lb.plantarum_K25GM002840	Methionine synthase II	0.514
Lb.plantarum_K25GM000706	Peptide ABC transporter ATP-binding protein	0.498
Lb.plantarum_K25GM002285	Carbamoyl-phosphate synthase small subunit	0.475
Lb.plantarum_K25GM002284	Carbamoyl phosphate synthase large subunit	0.448
Lb.plantarum_K25GM000682	Argininosuccinate synthase	0.43

Lipid transport and metabolism		
Protein-ID	NCBInr Description	Ratio
Lb.plantarum_K25GM000756	Acyltransferase	2.054
Lb.plantarum_K25GM001818	Short-chain dehydrogenase	1.848
Lb.plantarum_K25GM001393	3-oxoacyl-ACP synthase III	0.582
Lb.plantarum_K25GM001400	Acetyl-CoA carboxylase	0.549
Lb.plantarum_K25GM001397	3-oxoacyl-(acyl-carrier-protein) synthase II	0.539
Lb.plantarum_K25GM001403	Enoyl-[acyl-carrier-protein] reductase	0.51
Lb.plantarum_K25GM001395	ACP S-malonyltransferase	0.509
Lb.plantarum_K25GM001396	3-oxoacyl-ACP reductase	0.507
Lb.plantarum_K25GM001392	Beta-hydroxyacyl-ACP dehydratase	0.494
Lb.plantarum_K25GM001402	Acetyl-CoA carboxylase carboxyl transferase subunit alpha	0.491
Lb.plantarum_K25GM001399	Beta-hydroxyacyl-ACP dehydratase	0.477
Lb.plantarum_K25GM001401	Acetyl-CoA carboxylase subunit beta	0.476
Lb.plantarum_K25GM001394	Acyl carrier protein	0.46
Lb.plantarum_K25GM001398	Acetyl-CoA carboxylase biotin carboxyl carrier protein subunit	0.349

Cell wall/membrane/envelope biogenesis		
Protein-ID	NCBInr Description	Ratio
Lb.plantarum_K25GM001083	D-alanyl-D-alanine carboxypeptidase	3.075
Lb.plantarum_K25GM001081	Glycosyl transferase	2.333
Lb.plantarum_K25GM002062	UDP-N-acetylglucosamine 1-carboxyvinyltransferase	2.095
Lb.plantarum_K25GM000623	UDP-glucose 4-epimerase GalE	1.758
Lb.plantarum_K25GM002189	Sulfatase alkaline phosphatase superfamily membrane-bound protein	1.746
Lb.plantarum_K25GM000449	UDP-N-acetylmuramoyl-tripeptide--D-alanyl-D-alanine ligase	1.736
Lb.plantarum_K25GM003205	dTDP-4-dehydrorhamnose 3,5-epimerase	1.611
Lb.plantarum_K25GM002986	UDP-glucose 4-epimerase GalE	0.595

Chaperone		
Protein-ID	NCBInr Description	Ratio
Lb.plantarum_K25GM000048	Cold-shock protein A	3.258
Lb.plantarum_K25GM000090	Heat shock protein Hsp31	2.199
Lb.plantarum_K25GM001014	Cold-shock protein B	2.131
Lb.plantarum_K25GM000877	Cold shock protein CspC	2.108
Lb.plantarum_K25GM001703	Co-chaperone GrpE	0.652
Lb.plantarum_K25GM001594	ATP-dependent chaperone ClpB	0.543
Lb.plantarum_K25GM002894	Heat-shock protein Hsp20	0.519
Lb.plantarum_K25GM003063	ATP-dependent Clp protease ATP-binding subunit ClpL	0.509
Lb.plantarum_K25GM001702	Molecular chaperone DnaK	0.505
Lb.plantarum_K25GM000639	Molecular chaperone GroEL	0.498
Lb.plantarum_K25GM000638	Co-chaperone GroES	0.428

Transcription and Translation		
Protein-ID	NCBInr Description	Ratio
Lb.plantarum_K25GM001925	Methyltransferases	1.958
Lb.plantarum_K25GM002411	Methylated-DNA--[protein]-cysteine S-methyltransferase	3.591
Lb.plantarum_K25GM001549	tRNA (uracil-5-)-methyltransferase Gid	2.977
Lb.plantarum_K25GM001006	23S rRNA (uracil-5-)-methyltransferase RumA	2.759
Lb.plantarum_K25GM000615	Recombination protein RecR	2.741
Lb.plantarum_K25GM003341	Anaerobic ribonucleoside triphosphate reductase	2.463
Lb.plantarum_K25GM000678	Excinuclease ABC subunit B	2.432
Lb.plantarum_K25GM002761	Foldase	2.408
Lb.plantarum_K25GM001547	ATP-dependent protease subunit HslV	2.329
Lb.plantarum_K25GM000679	UvrABC system protein A	2.311
Lb.plantarum_K25GM001269	RNA-binding protein	2.158
Lb.plantarum_K25GM001347	16S rRNA (cytosine(967)-C(5))-methyltransferase	2.1
Lb.plantarum_K25GM001581	Ribosomal large subunit pseudouridine synthase B	2.093
Lb.plantarum_K25GM001716	50S ribosomal protein L7/L12	2.02
Lb.plantarum_K25GM002004	DNA recombination/repair protein RecA	1.992
Lb.plantarum_K25GM001016	ATP-dependent RNA helicase	1.977
Lb.plantarum_K25GM002261	Ribonucleotide reductase assembly protein NrdI	1.923
Lb.plantarum_K25GM001926	30S ribosomal protein S14	1.92
Lb.plantarum_K25GM001931	RNA pseudouridine synthase	1.897
Lb.plantarum_K25GM001017	Helicase	1.885
Lb.plantarum_K25GM001546	ATP-dependent protease ATPase subunit HslU	1.882
Lb.plantarum_K25GM001228	Foldase	1.881
Lb.plantarum_K25GM002043	Recombinase RarA	1.877
Lb.plantarum_K25GM002278	Helicase-exonuclease AddAB subunit AddA	1.817
Lb.plantarum_K25GM003337	Ribonucleotide-diphosphate reductase subunit alpha	1.799
Lb.plantarum_K25GM001468	RNA methyltransferase	1.798
Lb.plantarum_K25GM002789	RNA methyltransferase	1.788

Lb.plantarum_K25GM000896	30S ribosomal protein S12	1.781
Lb.plantarum_K25GM001916	16S rRNA (cytosine(1402)-N(4))-methyltransferase	1.755
Lb.plantarum_K25GM001234	tRNA (guanine-N(7)-)-methyltransferase	1.75
Lb.plantarum_K25GM001257	Translation initiation factor IF-3	1.747
Lb.plantarum_K25GM000004	DNA topoisomerase IV subunit B	1.743
Lb.plantarum_K25GM001816	Recombinase RecJ	1.728
Lb.plantarum_K25GM001892	tRNA (5-methylaminomethyl-2-thiouridylate)-methyltransferase	1.714
Lb.plantarum_K25GM001715	Translation initiation factor IF-2	1.683
Lb.plantarum_K25GM001490	Pseudouridine synthase	1.676
Lb.plantarum_K25GM001720	DNA polymerase III subunit alpha	1.654
Lb.plantarum_K25GM000701	Ribonuclease R	1.651
Lb.plantarum_K25GM000473	Ribosome-associated heat shock protein	1.647
Lb.plantarum_K25GM001993	Holliday junction DNA helicase RuvB	1.641
Lb.plantarum_K25GM001986	ATP-dependent RNA helicase	1.623
Lb.plantarum_K25GM001878	Ribonuclease J	1.619
Lb.plantarum_K25GM001117	16S rRNA pseudouridine(516) synthase	1.59
Lb.plantarum_K25GM001354	50S ribosomal protein L28	1.574
Lb.plantarum_K25GM000003	DNA recombination protein RecF	1.555
Lb.plantarum_K25GM000410	Ribosomal RNA small subunit methyltransferase A	1.548
Lb.plantarum_K25GM002279	ATP-dependent helicase/deoxyribonuclease subunit B	1.536
Lb.plantarum_K25GM001543	DNA topoisomerase IV subunit B	1.533
Lb.plantarum_K25GM000531	23S rRNA (guanosine(2251)-2'-O)-methyltransferase RlmB	1.5
Lb.plantarum_K25GM003348	Adenosine deaminase	0.616
Lb.plantarum_K25GM000233	Nucleoside-diphosphate kinase	0.574
Lb.plantarum_K25GM003353	DNA topoisomerase III	0.564
Lb.plantarum_K25GM000845	Asparagine--tRNA ligase	0.559
Lb.plantarum_K25GM003247	Topoisomerase I	0.508
Lb.plantarum_K25GM000558	DNA recombination protein RecT	0.506
Lb.plantarum_K25GM002283	Dihydroorotate dehydrogenase	0.451

Transcription Regulator		
Protein-ID	NCBInr Description	Ratio
Lb.plantarum_K25GM002204	Transcriptional regulator, RpiR family	1.871
Lb.plantarum_K25GM002319	GntR family transcriptional regulator	2.862
Lb.plantarum_K25GM000755	Bifunctional protein BirA	2.385
Lb.plantarum_K25GM000304	GntR family transcriptional regulator	1.777
Lb.plantarum_K25GM002638	TetR family transcriptional regulator	1.651
Lb.plantarum_K25GM001469	transcriptional regulator	3.258
Lb.plantarum_K25GM001322	TetR family transcriptional regulator	3.071
Lb.plantarum_K25GM001822	Transcriptional regulator, DeoR family	1.708
Lb.plantarum_K25GM000977	LysR family transcriptional regulator	2.108
Lb.plantarum_K25GM002251	GntR family transcriptional regulator	2.38
Lb.plantarum_K25GM002976	HTH-type transcriptional regulator GanR	2.131

Lb.plantarum_K25GM003058	ArsR family transcriptional regulator	1.716
Lb.plantarum_K25GM001616	HTH-type transcriptional repressor GlcR	0.611
Lb.plantarum_K25GM001030	Uncharacterized HTH-type transcriptional regulator YobD	0.599
Lb.plantarum_K25GM000243	Trehalose operon repressor	0.488
Lb.plantarum_K25GM000659	Stress-responsive transcriptional regulator	0.544
Lb.plantarum_K25GM001290	MarR family transcriptional regulator	0.478
Lb.plantarum_K25GM000173	Transcriptional regulator, LacI family	0.422
Lb.plantarum_K25GM001704	HrcA family transcriptional regulator	0.446
Lb.plantarum_K25GM001704	HrcA family transcriptional regulator	0.442

Signaling System		
Protein-ID	NCBInr Description	Ratio
Lb.plantarum_K25GM002758	Two-component sensor histidine kinase	1.676
Lb.plantarum_K25GM002561	ABC transporter ATP-binding protein/permease	2.012
Lb.plantarum_K25GM001558	Luciferase	1.959
Lb.plantarum_K25GM003089	Signal peptidase I	1.904
Lb.plantarum_K25GM001458	Metal ABC transporter substrate-binding protein	1.85
Lb.plantarum_K25GM000887	Cytochrome C2	1.731
Lb.plantarum_K25GM000346	ABC transporter ATP-binding protein	1.675
Lb.plantarum_K25GM000345	Glycine/betaine ABC transporter permease	1.508
Lb.plantarum_K25GM000648	Preprotein translocase subunit SecA	1.501
Lb.plantarum_K25GM000176	Sugar ABC transporter substrate-binding protein	0.494
Lb.plantarum_K25GM000181	Multiple sugar-binding transport ATP-binding protein MsmK	0.446
Lb.plantarum_K25GM002758	Two-component sensor histidine kinase	1.876

Antioxidant		
Protein-ID	NCBInr Description	Ratio
Lb.plantarum_K25GM002951	Thiol reductase thioredoxin	2.676
Lb.plantarum_K25GM001301	5-formyltetrahydrofolate cyclo-ligase	2.088
Lb.plantarum_K25GM001979	Thiol reductase thioredoxin	1.842
Lb.plantarum_K25GM003129	Ferrochelatase	1.776
Lb.plantarum_K25GM001208	Riboflavin biosynthesis protein RibD	0.602
Lb.plantarum_K25GM003057	Catalase	0.599
Lb.plantarum_K25GM003168	DNA-binding ferritin-like protein	0.48
Lb.plantarum_K25GM001243	Fe-S cluster assembly protein SufD	0.452
Lb.plantarum_K25GM001246	Fe-S cluster assembly protein SufB	0.394
Lb.plantarum_K25GM002951	Thiol reductase thioredoxin	2.676
Lb.plantarum_K25GM001301	5-formyltetrahydrofolate cyclo-ligase	2.088
Lb.plantarum_K25GM001979	Thiol reductase thioredoxin	1.842

Table. S2. The representative differential protein species in L.plantarum K25 between 10℃ and 37℃.
